# Impaired 11β-Hydroxysteroid Dehydrogenase Type 2 Activity in Kidney Disease Disrupts 11-Oxygenated Androgen Biosynthesis

**DOI:** 10.1210/clinem/dgae714

**Published:** 2024-10-09

**Authors:** Maria Tomkins, Tara McDonnell, Leanne Cussen, Michael S Sagmeister, Imken Oestlund, Fozia Shaheen, Lorraine Harper, Rowan S Hardy, Angela E Taylor, Lorna C Gilligan, Wiebke Arlt, Marie McIlroy, Declan de Freitas, Peter Conlon, Colm Magee, Mark Denton, Conall O’Seaghdha, Jacky L Snoep, Karl-Heinz Storbeck, Mark Sherlock, Michael W O’Reilly

**Affiliations:** Androgens in Health and Disease Research Group, Academic Division of Endocrinology, Department of Medicine, Royal College of Surgeons in Ireland, Dublin, D09 V2N0, Ireland; Department of Endocrinology, Beaumont Hospital, Dublin, D09 V2N0, Ireland; Androgens in Health and Disease Research Group, Academic Division of Endocrinology, Department of Medicine, Royal College of Surgeons in Ireland, Dublin, D09 V2N0, Ireland; Department of Endocrinology, Beaumont Hospital, Dublin, D09 V2N0, Ireland; Androgens in Health and Disease Research Group, Academic Division of Endocrinology, Department of Medicine, Royal College of Surgeons in Ireland, Dublin, D09 V2N0, Ireland; Department of Endocrinology, Beaumont Hospital, Dublin, D09 V2N0, Ireland; Steroid Metabolome Analysis Core (SMAC), Institute of Metabolism and Systems Research, University of Birmingham, Birmingham, B15 2TT, UK; Department of Nephrology, Institute of Applied Health Research, University of Birmingham, Birmingham, B15 2TT, UK; Department of Biochemistry, Stellenbosch University, Stellenbosch, 7600, South Africa; Steroid Metabolome Analysis Core (SMAC), Institute of Metabolism and Systems Research, University of Birmingham, Birmingham, B15 2TT, UK; Department of Nephrology, Institute of Applied Health Research, University of Birmingham, Birmingham, B15 2TT, UK; Institute of Clinical Sciences, University of Birmingham, Birmingham, B15 2TT, UK; Steroid Metabolome Analysis Core (SMAC), Institute of Metabolism and Systems Research, University of Birmingham, Birmingham, B15 2TT, UK; Steroid Metabolome Analysis Core (SMAC), Institute of Metabolism and Systems Research, University of Birmingham, Birmingham, B15 2TT, UK; Steroid Metabolome Analysis Core (SMAC), Institute of Metabolism and Systems Research, University of Birmingham, Birmingham, B15 2TT, UK; Medical Research Council Laboratory of Medical Sciences, London, W12 0HS, UK; Institute of Clinical Sciences, Imperial College London, London, SW7 2AZ, UK; Androgens in Health and Disease Research Group, Academic Division of Endocrinology, Department of Medicine, Royal College of Surgeons in Ireland, Dublin, D09 V2N0, Ireland; Department of Surgery, RCSI University of Medicine and Health Sciences, Dublin, D02 YN77, Ireland; Department of Nephrology, Beaumont Hospital/RCSI, Dublin, D09 V2N0, Ireland; Department of Nephrology, Beaumont Hospital/RCSI, Dublin, D09 V2N0, Ireland; Department of Nephrology, Beaumont Hospital/RCSI, Dublin, D09 V2N0, Ireland; Department of Nephrology, Beaumont Hospital/RCSI, Dublin, D09 V2N0, Ireland; Department of Nephrology, Beaumont Hospital/RCSI, Dublin, D09 V2N0, Ireland; Department of Biochemistry, Stellenbosch University, Stellenbosch, 7600, South Africa; Molecular Cell Biology, Vrije Universiteit Amsterdam, Amsterdam, 1081 HV, The Netherlands; Department of Biochemistry, Stellenbosch University, Stellenbosch, 7600, South Africa; Medical Research Council Laboratory of Medical Sciences, London, W12 0HS, UK; Androgens in Health and Disease Research Group, Academic Division of Endocrinology, Department of Medicine, Royal College of Surgeons in Ireland, Dublin, D09 V2N0, Ireland; Department of Endocrinology, Beaumont Hospital, Dublin, D09 V2N0, Ireland; Androgens in Health and Disease Research Group, Academic Division of Endocrinology, Department of Medicine, Royal College of Surgeons in Ireland, Dublin, D09 V2N0, Ireland; Department of Endocrinology, Beaumont Hospital, Dublin, D09 V2N0, Ireland

**Keywords:** 11-oxygenated androgens, steroid biosynthesis, chronic kidney disease, 11β-hydroxysteroid dehydrogenase type 2

## Abstract

**Context:**

11-Oxygenated androgens are a group of adrenal-derived steroids that require peripheral activation. In vitro data highlight a putative role for 11β-hydroxysteroid dehydrogenase type 2 (HSD11B2) in 11-oxygenated androgen biosynthesis, converting 11β-hydroxyandrostenedione to 11-ketoandrostenedione (11KA4), the direct precursor of the potent androgen 11-ketotestosterone (11KT). As the kidney is the major site of HSD11B2 expression, we hypothesized that patients with chronic kidney disease (CKD) would have reduced 11-oxygenated androgen biosynthesis due to impaired HSD11B2 activity.

**Objective:**

To determine the role of HSD11B2 in 11-oxygenated androgen biosynthesis using a human CKD cohort alongside complementary cell culture and computational modeling approaches.

**Methods:**

Cross-sectional observational study of patients with CKD (n = 85) and healthy controls (n = 46) measuring serum and urinary concentrations of glucocorticoids, and classic and 11-oxygenated androgens by liquid chromatography tandem mass spectrometry. A computational model of peripheral 11-oxygenated androgen biosynthesis was fitted to the serum data to calculate relative HSD11B2 expression levels for each participant.

**Results:**

HSD11B2 activity declined with estimated glomerular filtration rate (eGFR), evidenced by higher cortisol/cortisone (E) ratios in patients with CKD than in controls (*P* < .0001). Serum concentrations of E, 11KA4, 11KT, and 11β-hydroxytestosterone were lower in patients with CKD than in controls (*P* < .0001 for each). A computational model based on enzyme kinetic parameters of HSD11B2, 11β-hydroxysteroid dehydrogenase type 1, 17β-hydroxysteroid dehydrogenase type 2, and aldo-keto reductase 1C3 confirmed HSD11B2 as the key enzyme responsible for reduced 11-oxygenated androgen biosynthesis in CKD. Predicted HSD11B2 expression correlated with eGFR.

**Conclusion:**

This is the first in vivo study to confirm a central role for renal HSD11B2 in 11-oxygenated androgen biosynthesis. Determining the clinical implications of this observation for patients with CKD requires further research.

For decades the adrenal-derived C19 steroid 11β-hydroxyandrostenedione (11OHA4) was presumed to lack biological significance in humans. However, recent data and improved assay techniques have identified 11OHA4 as a key precursor of potent 11-oxygenated androgens that bind and activate the androgen receptor with similar potency to testosterone (T), with potentially significant roles in human health and disease (1-3). In contrast to classic adrenal and gonadal androgen secretion that declines with age, circulating concentrations of 11-oxygenated androgens remain relatively constant throughout life in both men and women (3-5). In recent years, research into 11-oxygenated androgens in disorders associated with androgen excess or androgen dependence such as polycystic ovary syndrome, congenital adrenal hyperplasia, premature adrenarche, and castration-resistant prostate cancer has highlighted the important and previously under-recognized role these androgens may play in human disease pathogenesis (3, 6-9).

11-Oxygenated androgen biosynthesis is initiated by the 11β-hydroxylation of adrenal-derived androstenedione (A4) by the exclusively adrenally expressed enzyme cytochrome P450 11β-hydroxylase, yielding 11OHA4 (Fig. 1A) (10). In vitro data suggest that 11OHA4 is converted to 11-ketoandrostenedione (11KA4) by the action of 11β-hydroxysteroid dehydrogenase type 2 (HSD11B2), which is predominantly expressed in mineralocorticoid-dependent tissues such as the kidney (11-13). Recent research has highlighted the potential role of the kidney in 11-oxygenated androgen biosynthesis by confirming renal HSD11B2 expression in the collecting duct and principal cells of the kidney with single cell RNA sequencing; concentrations of 11KA4 and 11-ketotestosterone (11KT) measured by liquid chromatography tandem mass spectrometry were also highest in the kidney compared with 6 other tissues (bladder, adipose, liver, intestine, prostate, skin) (14). However, to date this proposed catalytic step by HSD11B2 has not been explicitly confirmed in vivo. 11KA4 is subsequently activated in peripheral tissues such as adipose to 11KT by aldo-keto reductase type 1C3 (AKR1C3) (15, 16). 11KT and its 5α-reduced derivative 11-ketodihydrotestosterone bind and activate the androgen receptor with equivalent affinity and potency to their corresponding classic androgen counterparts, T and 5α-dihydrotestosterone (DHT), respectively (12, 17). Adipose, other glucocorticoid target tissues, and the liver express 11β-hydroxysteroid dehydrogenase type 1 (HSD11B1), which converts 11KA4 to 11OHA4 and inactivates 11KT to the less potent androgen 11β-hydroxytestosterone (11OHT) (15). Importantly, 11OHA4 is not a substrate for AKR1C3; therefore, the primary circulating pool of the active 11-oxygenated androgens, 11KT and 11OHT, are generated in peripheral tissues. Consequently, the conversion of 11OHA4 to 11KA4 by HSD11B2 has been proposed to be a critical step in the biosynthesis of active 11-oxygenated androgens (3, 11).

**Figure 1.**
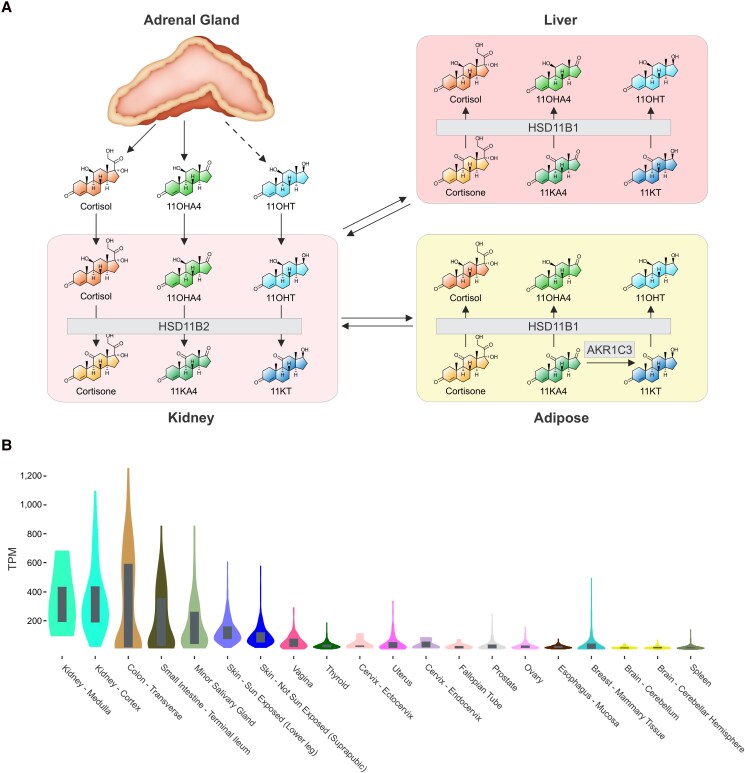
(A) Schematic overview of 11-oxygenated androgen biosynthesis. Cortisol, 11β-hydroxyandrostenedione (11OHA4) and low levels of 11β-hydroxytestosterone (11OHT) are produced in the adrenal gland and subsequently converted to cortisone, 11-ketoandrostenedione (11KA4), and 11-ketotestosterone (11KT) in the mineralocorticoid target tissue kidney by 11β-hydroxysteroid dehydrogenase type 2 (HSD11B2). The enzyme aldo-keto reductase type 1C3 (AKR1C3) facilitates the conversion of 11KA4 to the potent 11-oxygenated androgen 11KT in androgen target tissues such as adipose. Cortisone, 11KA4, and 11KT are converted back to cortisol, 11OHA4, and 11OHT by 11β-hydroxysteroid dehydrogenase type 1 (HSD11B1) expressed both in peripheral tissues and the liver. (B) Bulk tissue gene expression of HSD11B2 in human tissues. The kidney is the primary site of HSD11B2 gene expression. TPM, transcripts per million.

Chronic kidney disease (CKD) is an irreversible, progressive condition associated with excess cardiovascular morbidity and mortality, with a reported prevalence of 9% to 16% (18). The kidney is the primary site of HSD11B2 expression and activity in humans (Fig. 1B), with significant expression also found in the colon and the salivary glands. HSD11B2 has traditionally been viewed as a critical enzyme only in the context of glucocorticoid metabolism, converting cortisol (F) to cortisone (E), thereby protecting the mineralocorticoid receptor from excessive activation by high circulating concentrations of F (19-21). Reduced activity and expression of HSD11B2 have been reported in patients with CKD, with consequent perturbations in glucocorticoid metabolism (22-26). To date, confirmation of the role of HSD11B2 in 11-oxygenated androgen metabolism is limited to in vitro studies and has not been confirmed in carefully phenotyped human in vivo studies. Therefore, no data exist on the potential impact of CKD-related HSD11B2 dysfunction on peripheral 11-oxygenated androgen biosynthesis.

We hypothesized that patients with CKD would have dysfunctional 11-oxygenated androgen biosynthesis as a consequence of impaired renal HSD11B2 activity, leading to inefficient conversion of 11OHA4 to 11KA4, the substrate for AKR1C3-mediated 11-oxygenated androgen activation. This would provide in vivo confirmation of in vitro findings which suggest a pivotal role for HSD11B2 activity in 11-oxygenated androgen biosynthesis. We aimed to test this hypothesis by comprehensively profiling 11-oxygenated and classic androgens in a cohort of patients with CKD compared with a healthy control group. We also used enzyme assays in a cell culture model to determine the kinetics for relevant steroid substrates. Finally, we utilized a computational modeling approach to simulate the impact of decreased HSD11B2 activity across each stage of CKD on changes in circulating 11-oxygenated androgen metabolites.

## Materials and Methods

### Study Population

Patients with CKD were recruited consecutively from nephrology outpatient services in Beaumont Hospital, Ireland (n = 68) and University Hospital Birmingham, United Kingdom (n = 17). Eligibility criteria included patients ≥18 years of age with stable CKD. Participants with recent or current glucocorticoid therapy, mineralocorticoid therapy, or mineralocorticoid receptor antagonist use were excluded. Control participants had no history of kidney disease or other major chronic illness. Other exclusion criteria included pregnancy, recent use of glucocorticoid therapy within the last 3 months, use of the combined oral contraceptive pill, or drugs known to impact on steroid metabolism or on cytochrome P450 enzyme function.

### Study Design and Protocol

The study is a cross-sectional observational study where, following screening for eligibility and obtaining consent, participants attended the research unit for a single study visit after an 8-hour fast. Patients with CKD undergoing hemodialysis attended prior to a dialysis session. Patients using peritoneal dialysis attended on the morning of a peritoneal equilibration test following a standardized overnight dwell (2.27% glucose w/v solution).

The participants collected 24-hour urine samples in the home setting 48 hours prior to the study visit. The total 24-hour urine collection volume was recorded and a 10-mL sample was preserved for storage at −80 °C for urinary steroid metabolite excretion analysis at a later date; in-house analysis for urinary creatinine clearance and albumin–creatinine ratio was carried out on the day of study participation. Baseline bloods were drawn before 11 Am in the fasted state. Serum samples for multisteroid profiling were centrifuged and stored at −80 °C until analysis could be performed. Laboratory investigations included serum renal/bone profiles, fasting total cholesterol, high-density lipoprotein- and low-density lipoprotein-cholesterol, triglycerides, hemoglobin A1c, insulin, glucose, C-reactive protein, and a full blood count using in-hospital assays. Estimated glomerular filtration rate (eGFR) was calculated using the CKD Epidemiology Collaboration (CKD-EPI) 2021 Creatinine calculation (27). CKD stage was classified as CKD stage 3 for patients with an eGFR between 30 and 59 mL/min, CKD stage 4 for patients with an eGFR between 15 and 29 mL/min and CKD stage 5 for patients with an eGFR less than 15 mL/min or patients attending for dialysis (28). Anthropomorphic measurements at the study visit included height, weight, body mass index (BMI), and body composition as assessed by bioimpedance with Tanita MC-780 MA S and Tanita DC-360, recording measures for total, visceral, and free fat mass as well as muscle mass.

### Steroid Analysis

Serum and 24-hour urine samples for determination of circulating steroids and 24-hour urinary steroid metabolite excretion, respectively, were analyzed in batches. Steroids were extracted from 200 μL of serum or 24-hour urine and quantified by ultrahigh-performance liquid chromatography tandem mass spectrometry using a Waters Acquity ultrahigh-performance liquid chromatograph coupled to a Waters Xevo-XS triple quadrupole mass spectrometer, as previously described (29, 30).

For serum, a Phenomenex Luna omega 1.6 μm C18 2.1× 50 mm column was used with a methanol and water (0.1% formic acid) linear gradient system starting at 45% methanol and increasing to 75% methanol over 5 minutes, with post-column infusion of 6 mM ammonium fluoride at 5 μL/min. Calibration series ranged from 0.02 to 250 ng/mL.

For urine, steroids were deconjugated using a hydrolysis solution 200 μL of 0.2 M acetate buffer with 0.6 mg ascorbate and 0.6 mg of sulfatase (adjusted based on batch activity) added to each sample. This was heated at 60 °C for 3 hours to deconjugate steroids (31). Deconjugated steroids were extracted from the hydrolysis solution via solid phase extraction using a Biotage 100 mg C18 96 well extraction plate. A Waters HSS T3, 1.8 µm, 1.2 × 50 mm column was used with a methanol (0.1% formic acid) and water (0.1% formic acid) linear gradient starting at 30% methanol and increasing to 70% methanol over 16 minutes. Calibration series ranged from 0.5 to 6000 ng/mL.

The ratios of serum F/E and urinary F/E were used as a reflection of HSD11B2 activity in glucocorticoid inactivation. Calculated ratios using serum steroids 11OHA4/(11KA4 + 11OHT + 11KT) and urinary steroid metabolites (11β-hydroxyandrosterone [11βOHAn]/11-oxoandrosterone [11oxoAn]) were used to reflect the contribution of HSD11B2 to the biosynthesis of 11-oxygenated androgens.

The Beaumont Hospital and the previously published University of Birmingham control cohorts were analyzed using different steroid extraction methods. It was recently reported that heating during the drying of steroids results in the thermal degradation of F and E to 11OHA4 and 11KA4, respectively, thus leading to the overestimation of these steroid levels (32). As the CKD cohort and the Beaumont control cohort were extracted without the application of heat during drying and analyzed on the same platform at the same time, we used the Beaumont control cohort as our primary comparator group in the “Results” as well as in figures and tables. However to control for the impact of the age discrepancy between the CKD and Beaumont controls, we used 2 approaches. Firstly, we analyzed the older aged-matched University of Birmingham cohort as a second comparator group (see Tables 1 and 2). Importantly we excluded 11OHA4 and 11KA4 from this analysis as these steroids are subject to overestimation with heated drying methods. Secondly, we used a multiple linear regression model to control for the confounding impact of age on analysis and comparison in the Beaumont control and CKD cohorts.

**Table 1.** Baseline characteristics of the study population

	CKD (n = 85)	Beaumont Hospital control (n = 46)	University of Birmingham control (n = 153)
**Baseline Demographics**			
Age (years)	64 (52-71)	34 (31-66)*^a^*	63 (52-74)
Sex, n (%) male	55 (65%)	8 (17%)*^a^*	93 (61%)
Body mass index (kg/m^2^)	27.4 (23.9-31.0)	25.8 (22.4-30.3)	Not available
eGFR (mL/min)	22.0 (13.0-38.0)	103.0 (90.0-119.0)*^a^*	Not available
UACR (mg/mmol)	19.2 (5.0-96.2)	Not available	Not available
CKD stage, n (%)		Not applicable	Not applicable
3	30 (35%)		
4	28 (33%)		
5	27 (32%)		
Cause of CKD, n (%)		Not applicable	Not applicable
Glomerulonephritis	22 (26%)		
Hereditary/Congenital	11 (13%)		
Unknown	9 (11%)		
HTN	9 (11%)		
Nephrectomy/Unilateral kidney	8 (9%)		
Interstitial nephritis	8 (9%)		
Obstructive uropathy	7 (8%)		
Renovascular disease	4 (5%)		
Infiltrative (sarcoid, amyloid)	3 (4%)		
Other (UTI, hypercalcemia)	2 (2%)		
Diabetic nephropathy	2 (2%)		

Data presented as median (IQR) unless otherwise stated.

Abbreviations: CKD, chronic kidney disease; eGFR, estimated glomerular filtration rate; HTN, hypertension; UACR, urine albumin to creatinine ratio; UTI, urinary tract infection.

^*a*^ *P* < .0001 for the comparison CKD vs Beaumont Hospital controls.

**Table 2.** Steroid concentrations and ratios between the study cohorts

	CKD (n = 85)	Beaumont Hospital control (n = 46)	University of Birmingham control (n = 153)
**Classic androgens**			
*Serum*			
DHEA (nmol/L)			
All participants	6.3 (3.9-9.3)	13.8 (9.6-17.1)*^d^*	5.1 (3.3-8.4)*^e^*
Male	6.0 (3.9-8.5)	6.8 (5.4-10.2)	5.1 (3.0-8.1)
Female	7.4 (4.1-10.7)	14.0 (11.3-18.4)*^d^*	5.2 (3.5-8.6)
A4)(nmol/L)			
All participants	1.9 (1.1-2.8)	3.3 (2.4-4.7)*^d^*	1.4 (1.0-2.1)
Male	1.9 (1.2-2.8)	3.4 (3.1-5.1)*^d^*	1.5 (1.1-2.1)
Female	2.5 (0.9-3.1)	3.2 (2.4-4.5)*^c^*	1.3 (0.7-2.2)*^e^*
Testosterone (nmol/L)			
Male	15.5 (12.2-19.4)	19.1 (12.2-27.0)	11.7 (9.3-15.3)*^g^*
Female	0.8 (0.5-1.3)	1.0 (0.8-1.2)	0.5 (0.2-0.7)*^g^*
DHT (nmol/L)			
Male	1.5 (0.8-2.0)	1.5 (0.7-2.5)	1.2 (0.8-1.5)
Female	0.2 (0.2-0.4)	0.4 (0.2-0.5)*^a^*	0.2 (0.2-0.2)
*Urine*			
Androsterone (μg/24 hours)			
All participants	1022 (489-2227)	1408 (807-2104)	Not available
Male	1613 (649-2520)	1197 (802-1457)	Not available
Female	693 (343-1215)	1422 (804-2260)*^b^*	Not available
Etiocholanolone (μg/24 hours)			
All participants	1177 (673-2215)	1638 (1103-2506)*^a^*	Not available
Male	1488 (741-2546)	1351 (710-1971)	Not available
Female	811 (391-1652)	1713 (1161-2703)*^c^*	Not available
DHEA (μg/24 hours)			
All participants	12.5 (12.5-46.5)	36.1 (13.6-68.1)*^b^*	Not available
Male	12.5 (12.5-47.3)	12.5 (12.5-23.2)	Not available
Female	12.5 (12.5-40.1)	41.3 (12.5-80.4)*^b^*	Not available
**11-oxygenated androgens**			
*Serum*			
11OHA4 (nmol/L)			
All participants	5.5 (3.7-7.6)	4.0 (3.4-5.5)*^a^*	Not available
Male	6.2 (3.8-8.9)	Not available	Not available
Female	6.1 (4.0-8.6)	4.0 (3.4-5.5)*^b^*	Not available
11KA4 (nmol/L)			
All participants	0.6 (0.4-0.9)	1.0 (0.8-1.2)*^d^*	Not available
Male	0.7 (0.4-1.1)	Not available	Not available
Female	0.7 (0.4-0.9)	1.0 (0.8-1.2)*^d^*	Not available
11KT (nmol/L)			
All participants	0.5 (0.2-0.9)	1.3 (1.0-1.5)*^d^*	0.8 (0.6-1.2)*^h^*
Male	0.4 (0.2-0.7)	1.4 (0.8-2.0)*^d^*	0.8 (0.6-1.3)*^g^*
Female	0.5 (0.2-0.9)	1.3 (1.0-1.5)*^d^*	0.9 (0.6-1.1)*^f^*
11OHT (nmol/L)			
All participants	0.2 (0.2-0.5)	0.6 (0.5-0.8)*^d^*	0.6 (0.4-0.9)*^h^*
Male	0.2 (0.2-0.5)	0.7 (0.4-1.6)*^b^*	0.7 (0.4-0.9)*^h^*
Female	0.4 (0.2-0.7)	0.6 (0.5-0.8)*^b^*	0.5 (0.4-0.9)*^e^*
*Urine*			
11βOHAn (μg/24 hours)			
All participants	554 (323-990)	544 (405-758)	Not available
Male	550 (378-900)	741 (477-920)	Not available
Female	373 (185-565)	544 (405-758)*^b^*	Not available
11oxoAn (μg/24 hours)		52 (41-61)	
All participants	46 (24-73)	52.5 (31.3-70.5)	Not available
Male	43.2 (24.8-74.6)	52.0 (41.1-60.8)*^a^*	Not available
Female	41.0 (15.4-57.0)		Not available
**11β-Hydroxysteroid dehydrogenase 2 activity—steroid ratios**			
*Serum*			
Serum cortisol (F)/cortisone (E) ratio			
All participants	10.9 (8.1-14.0)	5.6 (4.5-7.1)*^d^*	6.7 (5.6-8.1)*^h^*
Male	10.7 (7.6-13.7)	6.2 (4.7-8.3)*^b^*	6.6 (5.6-8.3)*^h^*
Female	11.1 (8.8-14.3)	5.5 (4.5-6.9)*^d^*	6.9 (5.7-8.2)*^h^*
Serum 11OHA4/11KA4 ratio			
All participants	8.3 (6.7-11.6)	3.7 (3.0-4.7)*^d^*	Not available
Male	7.8 (6.3-11.5)	Not available	Not available
Female	8.7 (7.1-13.1)	3.7 (3.0-4.7)*^d^*	Not available
Serum 11OHA4/(11KA4 + 11KT + 11OHT) ratio			
All participants	3.9 (2.8-5.5)	1.5 (1.2-1.8)*^d^*	Not available
Male	3.9 (2.9-5.7)	Not available	Not available
Female	3.8 (2.7-5.1)	1.5 (1.2-1.8)*^d^*	Not available
*Urine*			
Urinary F/E ratio			
All participants	0.8 (0.7-1.0)	0.6 (0.5-0.7)*^d^*	Not available
Male	0.9 (0.7-1.0)	0.6 (0.5-0.7)*^c^*	Not available
Female	0.8 (0.6-0.9)	0.6 (0.5-0.7)*^b^*	Not available
Urinary 11βOHAn/11oxoAn ratio			
All participants	14.0 (9.9-17.9)	11.3 (8.6-14.3)*^a^*	Not available
Male	13.8 (10.1-17.9)	13.6 (10.3-18.2)	Not available
Female	14.4 (9.9-17.8)	11.0 (8.5-14.1)*^a^*	Not available

Data presented as median (IQR).

Abbreviations: 11βOHAn, 11β-hydroxyandrosterone; 11KA4, 11-ketoandrostenedione; 11KT, 11-ketotestosterone; 11OHA4, 11β-hydroxyandrostenedione; 11OHT, 11β-hydroxytestosterone; 11oxoAn, 11-oxoandrosterosterone; A4, androstenedione; CKD chronic kidney disease; DHEA, dehydroepiandrosterone; DHT, 5α-dihydrotestosterone; E, cortisone; F, cortisol.

^*a*^ *P* < .05 for comparison of CKD vs Beaumont controls.

^*b*^ *P* < .01 for comparison of CKD vs Beaumont controls.

^*c*^ *P* < .001 for comparison of CKD vs Beaumont controls.

^*d*^ *P* < .0001 for comparison of CKD vs Beaumont controls.

^*e*^ *P* < .05 for comparison of CKD vs University of Birmingham controls.

^*f*^ *P* < .01 for comparison of CKD vs University of Birmingham controls.

^*g*^ *P <* .001 for comparison of CKD vs University of Birmingham controls.

^*h*^ *P* < .0001 for comparison of CKD vs University of Birmingham controls.

### HSD11B2 Expression Analysis

HSD11B2 expression data (dbGaP Accession phs000424.v8.p2) was obtained from the Genotype-Tissue Expression (GTEx Portal) on January 30, 2024. The 20 tissue types with the highest median HSD11B2 expression values were ranked from highest to lowest and are shown in Fig. 1B.

### Determination of the Kinetic Parameters for HSD11B2

HEK293 cells were purchased from the American Type Culture Collections and cultured in high glucose Dulbecco's modified Eagle’s medium supplemented with 10% fetal bovine serum, 1.5 g/L NaHCO_3_ and 1% penicillin–streptomycin. Cells were maintained at 37 °C, 5% CO_2_, and in 90% humidity. Authentication was performed by short-tandem repeat profiling (NorthGene) and the cells were regularly tested for contamination by mycoplasma. HEK293 cells were plated into 10 cm^2^ tissue culture dishes at a concentration of 2 × 10^6^ live cells per dish. After 24 hours, cells were transfected with 10 µg of a pcDNA3.1(−) vector containing HSD11B2 plasmid (a gift from Prof. K. Chapman, University of Edinburgh, UK) using X-tremeGENE HP DNA transfection reagent (Roche). After 24 hours, transfected cells were replated into 48-well Corning CELLBIND plates at a concentration of 2 × 10^5^ cells per mL (300 µL per well). Cells were then incubated for an additional 24 hours to allow for attachment. Media were subsequently replaced with serum-free media containing steroid substrate (11OHA4, 11OHT, or F) at concentrations ranging from 0.1 µM to 10 µM. Aliquots (250 µL) were then collected at specific time intervals over the period of 48 hours to enable the plotting of progress curves. Steroids were extracted and quantified by ultrahigh-performance liquid chromatography tandem mass spectrometry as previously described (15). Michaelis–Menten type equations were used to construct ordinary differential equations to fit to the progress curves for estimation of apparent *V*_max_ and *K*_m_ values (see Supplementary information for details (33)). Conversion of 100 nM 11OHA4 to 11KA4 was included in all experiments and the conversion rate used to normalize the transfection efficiency between experiments, which was set to 1 for the reported parameter values. Activities are expressed as μM/min (units/well volume).

### Computational Modeling Methods

A mathematical model was constructed from experimentally parameterized rate equations for the individual enzymes (15, 16, 34), using the schema in Fig. 1A to define ordinary differential equations for each of the steroids. The full model description is provided elsewhere (Supplementary material (33)). The relative expression levels of the enzymes in the individual patients were estimated by minimizing the sum of the squared ordinary differential equations values (steady-state assumption), weighed according to the total concentrations of the respective metabolite groups (glucocorticoids, classic androgens, and 11-oxygenated androgens, respectively). For the minimization, a multiplier for the individual enzymes was fitted relative to the expression of AKR1C3, which was set to 1, and the expression levels for the healthy control group were used as reference values for the other simulations.

### Statistics

Normality of quantitative variables was tested with the Shapiro–Wilk test. The majority of data did not exhibit normal distribution and, accordingly, data are presented as median and interquartile range, unless otherwise stated. Where samples demonstrated a steroid concentration below the lower limit of accurate assay quantification, their value was substituted for half the lower limit of quantification for the purposes of statistical analysis. Samples with undetectable steroid concentrations (below the limit of detection) were included as 0. Correlations of eGFR and steroid hormone concentrations or steroid hormone ratios were evaluated using Spearman analysis and the relationship between stage of CKD and steroid concentration/ratio was analyzed using the Mann–Whitney, Kruskal–Wallis, and Dunn's multiple comparisons tests. Statistical significance was defined for *P* < .05. Multiple linear regression analysis on log-transformed values of each relevant classic and 11-oxygenated androgen was performed to further delineate the relationship between classic and 11-oxygenated androgen concentrations and eGFR when accounting for age, sex, and BMI. Statistical significance was defined for *P* < .012 using Bonferroni correction for the multiple linear regression. Statistical analysis was performed using R version 4.3.1 and GraphPad Prism version 10.2.3 for Windows, (GraphPad Software, San Diego, CA, USA).

### Study Approval

Institutional approval was obtained at all study sites for recruitment of patients with CKD as well as healthy controls to a number of metabolic phenotyping studies. Patients with CKD were recruited in Beaumont Hospital to the Corticosteroid metabolomics in Chronic Kidney Disease (CORT-CKD) study (Beaumont Hospital Research Ethics Committee reference 21/52). Additional patients with CKD and healthy controls were recruited at University Hospital Birmingham to the Steroid Hormone Metabolism and Muscle Loss in Chronic Kidney Disease (SMK) Study (London-Stanmore Research Ethics Committee reference 22/PR/0360; ISRCTN15497320). Healthy controls in Beaumont Hospital were recruited to the Femail Metabolic Risk and Androgens: an Irish Longitudinal (FEMAIL) Study (Beaumont Hospital Research Ethics Committee reference 20/49). Healthy controls were recruited via news bulletins at Beaumont Hospital/RCSI and the University of Birmingham (including the 1000 Elders cohort (35)). Further control participants from the University of Birmingham were available from healthy volunteers recruited to the Science, Technology, Engineering and Mathematics Ethical Review Committees of the University of Birmingham, UK (ERN_17-0494, ERN_17-0494B) and the Mayo Clinic, Rochester, MN, USA (IRB ID 18-009787) (5). All patients provided written informed consent prior to participation.

## Results

### Description of Cohorts

Baseline characteristics of the study population are summarized in Table 1. The CKD cohort consisted of 85 participants with a median age of 64 years (65% male), median eGFR of 22 mL/min, and median urine albumin–creatinine ratio of 19.2 mg/mmol. Patients with CKD were distributed across each stage of CKD (3, 4, and 5) at 35%, 33%, and 32%, respectively. Eleven patients (13%) were on dialysis, 8 attending for intermittent hemodialysis and 3 using automated peritoneal dialysis. Only 2% of participants had diabetes mellitus or diabetic nephropathy. The etiology of underlying CKD is outlined in Table 1. Control participants (n = 46, referred to as Beaumont controls) had a median age of 34 years, 17% were male, and all had normal renal function with a median eGFR of 103 mL/min. There were no significant differences in BMI between the CKD and control cohort; however, the control cohort was younger and had a significantly greater female preponderance. To account for these differences we also compared the steroid profiles in our CKD cohort with those from age- and sex-matched controls (n = 153, referred to as Birmingham controls) extracted from a previously published healthy control cohort (5) to demonstrate that age and sex were not factors affecting glucocorticoid or 11-oxygenated androgen levels in healthy individuals. Results and figures refer to the CKD and Beaumont control cohort. Additional analysis of the second control group is represented in Table 2, excluding data on 11OHA4 and 11KA4 for the reasons outlined above.

### HSD11B2 Activity is Impaired in CKD

**Figure 2.**
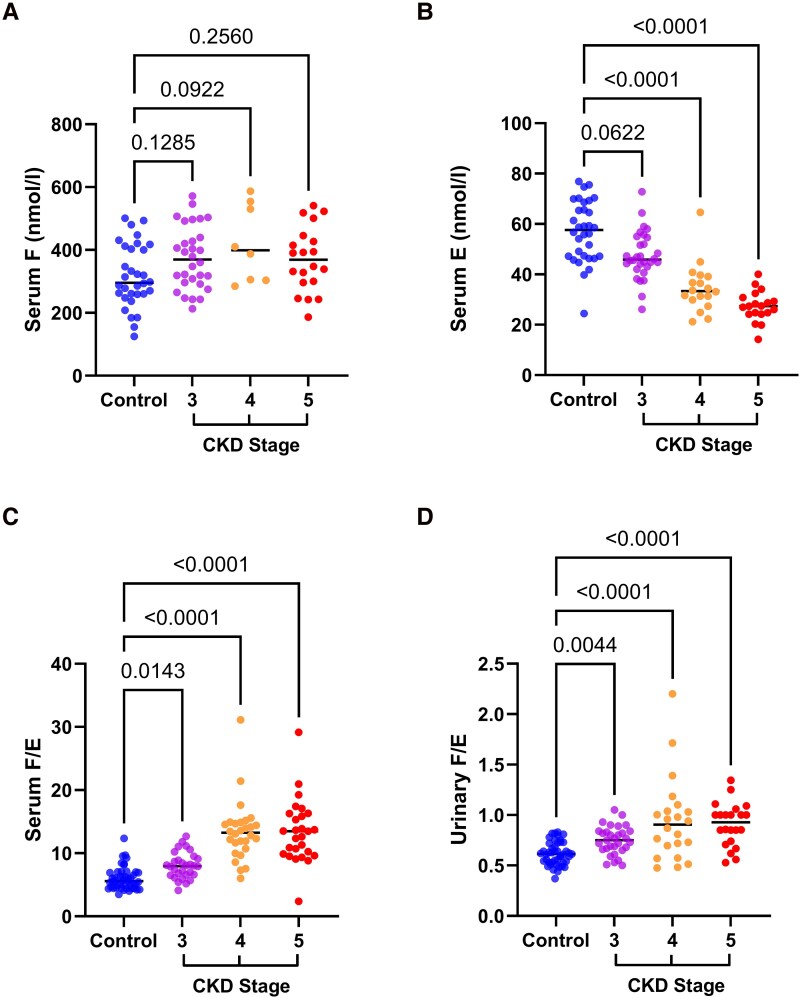
Reduced 11β-hydroxysteroid dehydrogenase type 2 activity in patients with CKD. (A) Serum cortisol (F) concentrations in controls compared with chronic kidney disease (CKD) stages 3, 4, and 5. (B) Serum cortisone (E) concentrations in controls compared with CKD stage 3, 4, and 5. (C) Serum F/E ratio in controls compared with CKD 3, 4, and 5. (D) Urinary F/E ratio in controls compared with CKD 3, 4, and 5.

The results of the differences in steroid concentrations and ratios between the cohorts are represented in Table 2. Participants with CKD had greater serum F concentrations than control participants (median serum F 393 [301-493] vs 296 [259-410] nmol/L, *P* = .002). There were no significant differences in serum F when stratified by CKD stage compared with control participants (Fig. 2A). Patients with CKD had significantly lower serum E than controls (37.5 [28.4-46.0] vs 57.6 [47.2-67.9] nmol/L, *P* < .0001), with a stepwise decline with increasing CKD stage on Dunn's multiple comparison test (Fig. 2B). Serum E correlated positively with eGFR (r = 0.76, *P* < .01). HSD11B2 activity, as measured by the serum F/E ratio, was significantly higher in patients with CKD than controls, indicative of reduced HSD11B2 activity in CKD (serum F/E ratio 10.9 vs 5.4, *P* < .0001). The serum F/E ratio increased as renal function declined when stratified by CKD stage on Dunn's multiple comparison test (Fig. 2C). eGFR and the serum F/E ratio were negatively correlated (r = −0.68, *P* < .01).

Urinary F was reduced in patients with CKD compared with controls (54.6 [42.8-78.5] vs 73.1 [47.1-88.0] μg/24 hours, *P* = .03). Urinary E was also reduced in patients with CKD compared with controls (73.1 [47.6-91.3] vs 113.9 [96.5-137.0] μg/24 hours, *P* < .0001). The urinary F/E ratio was increased in patients with CKD at 0.8 compared with 0.6 in control participants (*P* < .0001), consistent with impaired HSD11B2 activity in CKD. This ratio increased in a stepwise manner across each stage of CKD on Dunn's multiple comparison test (Fig. 2D).

There were no significant differences in 24-hour excretion of the predominant glucocorticoid metabolites 11β-hydroxyetiocholanolone or 11-oxoetiocholanolone in patients with CKD compared with controls (11β-hydroxyetiocholanolone 341.0 [170.0-564.5] vs 250.9 [156.4-348.4] μg/24 hours, respectively [*P* = .07], 111-oxoetiocholanolone 430.5 [225.3-686.9] vs 315.8 [203.5-468.0] μg/24 hours, respectively [*P* = .16)).

### 11-oxygenated Androgen Biosynthesis is Reduced in CKD Due to Reduced HSD11B2 Activity

**Figure 3.**
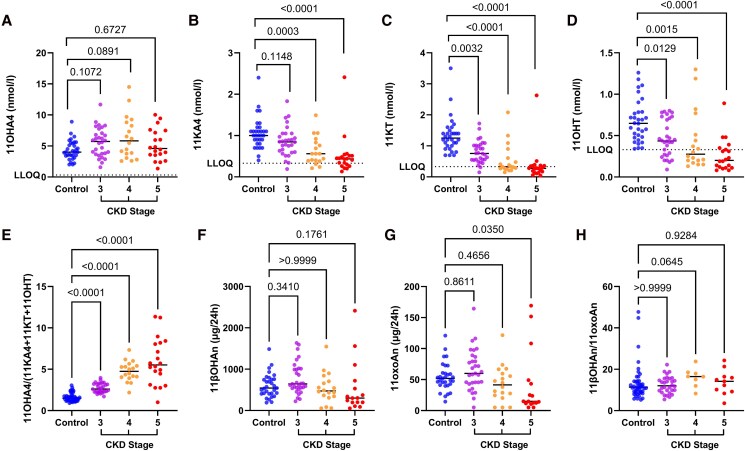
Reduced 11-oxygenated androgen biosynthesis in patients with chronic kidney disease (CKD) compared with controls. (A) Serum 11β-hydroxyandrostenedione (11OHA4) in controls compared with CKD stage 3, 4, and 5. (B) Serum 11-ketoandrostenedione (11KA4) in controls compared with CKD stage 3, 4, and 5. (C) Serum 11-ketotestosterone (11KT) concentrations in controls compared with CKD stage 3, 4, and 5. (D) Serum 11β-hydroxytestosterone (11OHT) concentrations in controls compared with CKD stage 3, 4, and 5. (E) Serum 11OHA4/(11KA4 + 11KT + 11OHT) ratio in controls compared with CKD stage 3, 4, and 5. (F) Urinary 11β-hydroxyandrosterone (11βOHAn) concentrations in controls compared with CKD stage 3, 4, and 5. (G) Urinary 11-oxoandrosterone (11oxoAn) concentrations in controls compared with CKD stage 3, 4, and 5. (H) Urinary 11βOHAn/11oxoAn ratio in controls compared with CKD stage 3, 4, and 5. LLOQ, lower limit of quantification.

Serum 11OHA4 concentrations were significantly elevated in patients with CKD compared with controls (*P* = .02, Table 2). There was no difference in 11OHA4 concentrations between the groups when stratified by CKD stage (Fig. 3A). Participants with CKD had lower serum concentrations of all 11-oxygenated androgens downstream of 11OHA4: serum 11KA4 (*P* < .0001), serum 11KT (*P* < .0001), and serum 11OHT (*P* < .0001) (Table 2). When stratified by CKD stage there was a stepwise decline in serum 11KA4, 11KT, and 11OHT (Fig. 3B-3D).

We calculated the serum ratio of 11OHA4/(11KA4 + 11OHT + 11KT) as a marker of HSD11B2 activity in the context of 11-oxygenated androgen biosynthesis. Here, 11OHA4 was considered as the substrate for HSD11B2, while 11KA4, 11OHT, and 11KT were all considered products. This ratio was increased in patients with CKD compared with controls (3.9 vs 1.5, *P* < .0001, Table 2), indicative of reduced HSD11B2 activity. Across each stage of CKD, this ratio demonstrated a stepwise increase indicative of an incremental loss of HSD11B2 activity and a reduction in downstream active 11-oxygenated androgens with progression of renal failure (Fig. 3E).

**Figure 4.**
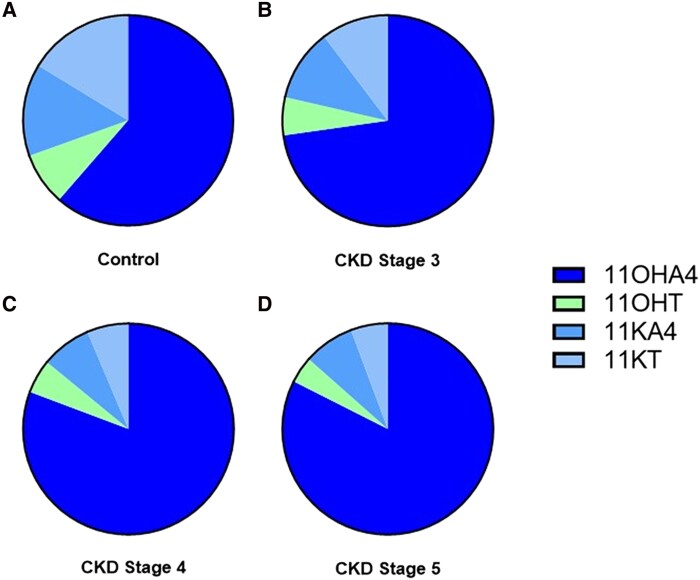
Relative contribution (%) of individual steroids (11OHA4, 11OHT, 11KA4, 11KT) to the total circulating 11-oxygenated androgen steroid pool in (A) Controls, (B) CKD Stage 3, (C) CKD Stage 4, and (D) CKD Stage 5.

Abbreviations: 11OHA4, 11β-hydroxyandrostenedione; 11OHT, 11β-hydroxytestosterone; 11KA4, 11-ketoandrostenedione; 11KT, 11-ketotestosterone.

We calculated the relative proportion of each 11-oxygenated androgen to the total circulating 11-oxygenated androgen pool in patients with CKD and control participants. There was no difference between median total 11-oxygenated androgen concentrations (11OHA4 + 11KA4 + 11KT + 11OHT) in patients with CKD compared with controls (6.9 [4.6-9.5] vs 7.0 [5.6-9.1] nmol/L, respectively, *P* = .62). Serum 11OHA4 had a greater proportional contribution to the total 11-oxygenated androgen pool in patients with CKD than in controls (77% [72-84] v 60% [55-64], *P* < .0001) (Fig. 4). In contrast, serum concentrations of 11KA4, 11KT, and 11OHT all had a proportionally lower contribution to the total 11-oxygenated androgen pool in patients with CKD than in controls (*P* < .0001 for each, Fig. 4).

There were no significant differences between the 2 cohorts in 24-hour urinary excretion of 11βOHAn (*P* = .70). When stratified by CKD stage, there was reduced 24-hour urinary excretion of 11βOHAn in patients with CKD compared with controls using the Kruskal–Wallis test (*P* = .01), with no discrete differences between controls and each CKD stage on Dunn's multiple comparison test (Fig. 3F). There was no significant difference in 24-hour urinary excretion of the 11-oxygenated androgen metabolite 11oxoAn in the CKD cohort compared with controls (*P* = .09, Table 2); when stratified by CKD stage using Kruskal–Wallis and Dunn's multiple comparison tests, there was reduced urinary excretion of 11oxoAn most pronounced in CKD stage 5 (Fig. 3G). The ratio of urinary 11βOHAn/11oxoAn was elevated in patients with CKD compared with controls (*P* < .01, Table 2).

### Classic Androgens in CKD

Serum concentrations of dehydroepiandrosterone (DHEA) were significantly lower in female patients (*P* < .0001) with CKD than in controls, with no significant difference reported in male patients (Table 2). Serum A4 was also lower in patients with CKD than in controls in males and females (*P* < .001 for both, Table 2). There was no significant difference in serum T between patients with CKD and controls. Serum DHT was lower in the female CKD cohort than in female controls (*P* = .02). When classic androgen concentrations in patients with CKD were compared with the age- and sex-matched Birmingham control cohort, differences in DHEA, A4, and DHT were less significant between the groups. Male patients with CKD had significantly higher serum T concentrations and female patients with CKD had significantly lower serum T concentrations than the Birmingham control cohort, although median levels remained within expected normal reference ranges.

Female patients with CKD had reduced 24-hour urinary concentrations of the classic androgen metabolites androsterone, etiocholanolone, and DHEA compared with controls (*P* = .002, *P* = .0007, *P* = .009, respectively). There were no differences in 24-hour urinary concentrations of the classic androgen metabolites in males (Table 2).

### Relationship of Serum 11-Oxygenated and Classic Androgens With Renal Function Using Linear Regression Analysis

Linear regression analysis of the CKD cohort and Beaumont control cohort was performed to account for differences in age and sex between the cohorts. In the absence of a linear relationship between serum 11OHA4 or serum T and eGFR, multivariate analysis was not performed on these steroids. 11KA4, 11KT, and 11OHT had a persistent, although weak, association with eGFR after controlling for age, sex, and BMI. A positive association was observed between serum 11OHT concentrations and age with no relationship found between 11OHT and either sex or BMI (Table S1 (33)). The association between DHEA and A4 with eGFR was not significant (*P* > .012) when counting for age, BMI, and sex using multivariate regression (Table S1 (33)).

### HSD11B2 Efficiently Catalyzes the Dehydrogenation of 11-Oxygenated Androgens

HSD11B2 was expressed in nonsteroidogenic HEK293 cells and assayed for activity to determine the kinetic parameters (*K*_m_ and *V*_max_). Rate equations with Michaelis–Menten type rate equations were fitted to the progress curves for 3 independent experiments as shown elsewhere (Fig. S2 (33)). The apparent *K*_ma_ (*K*_ms, app_) of HSD11B2 for F and 11OHA4 were similar (0.4 µM and 0.8 µM), while that for 11OHT (0.1 µM) was lower, indicating a higher substrate affinity for 11OHT (Table 3). The apparent *V*_max_ (*V*_max, app_) for 11OHT (0.08 µM/h) was however 6-fold lower than that of 11OHA4 (0.52 µM/h), but 3-fold higher than that of F (0.03 µM/h). The *V*_max_/*K*_ms_ values, an estimate of enzyme efficiency, show that both 11OHA4 (0.68) and 11OHT (0.8) were more efficiently converted by HSD11B2 than the better-known glucocorticoid substrate F (0.08) (Table 3).

**Table 3.** Kinetic constants (*K*_ms, app_; *K*_mp, app_; *V*_max, app_; *V*_max_/*K*_ms_) determined for HSD11B2

Reaction	*K*_ms, app_ (µM)	*K*_mp app_ (µM)	*V*_max, app_ (µM/h)	*V*_max_/*K*_ms_
Cortisol → Cortisone	0.38 (±0.02)		0.03 (±0.001)	0.08
*11β-hydroxyandrostenedione* → 11-ketoandrostenedione	0.77 (±0.07)	1.73 (±0.48)	0.52 (±0.04)	0.68
*11β−hydroxytestosterone* → 11-ketotestosterone	0.10 (±0.01)	0.31 (±0.18)	0.08 (±0.004)	0.8

Parameter values are given ± standard error.

Abbreviations: *K*_ms, app_, apparent Michaelis–Menten constant; *V*_max, app_, apparent maximum rate; *V*_max_/*K*_ms_, estimate of enzyme efficiency.

### Enzymatic Modelling Highlights a Central Role for HSD11B2 in 11-Oxygenated Androgen Metabolism

The above analysis for the individual reactions indicates that eGFR is correlated with HSD11B2 activity. We subsequently set out to test this hypothesis quantitatively by constructing a computational model to describe peripheral 11-oxygenated androgen biosynthesis as well as the peripheral interconversion of the glucocorticoids (F and E) and classic androgens (A4 and T). The model was based on the parameterized rate equations for the 4 main enzymes involved in the peripheral biosynthesis of 11-oxygenated androgens, namely HSD11B2, AKR1C3, HSD11B1 and 17β-hydroxysteroid dehydrogenase type 2 (HSD17B2). The kinetics for AKR1C3, HSD17B2, and HSD11B1 were all previously determined (15, 16, 34), while we determined the kinetics of HSD11B2 using the same methodology here. Using the circulating concentrations of the 11-oxygenated androgens, classic androgens and glucocorticoids determined for the control group, we assumed the metabolites to be in steady state and used the model to find the relative expression levels of the 4 enzymes that would account for the measured steroid levels. We used only the female CKD data sets as to eliminate the effect of differences in the overall circulating classic androgens between males and females and because the control group contained predominantly females. The expression of HSD11B2, HSD11B1, and HSD17B2 was set relative to AKR1C3. The relative expression levels of the 4 enzymes were identifiable in the control group, and these were subsequently used as reference values for testing the hypothesis in the CKD groups.

**Figure 5.**
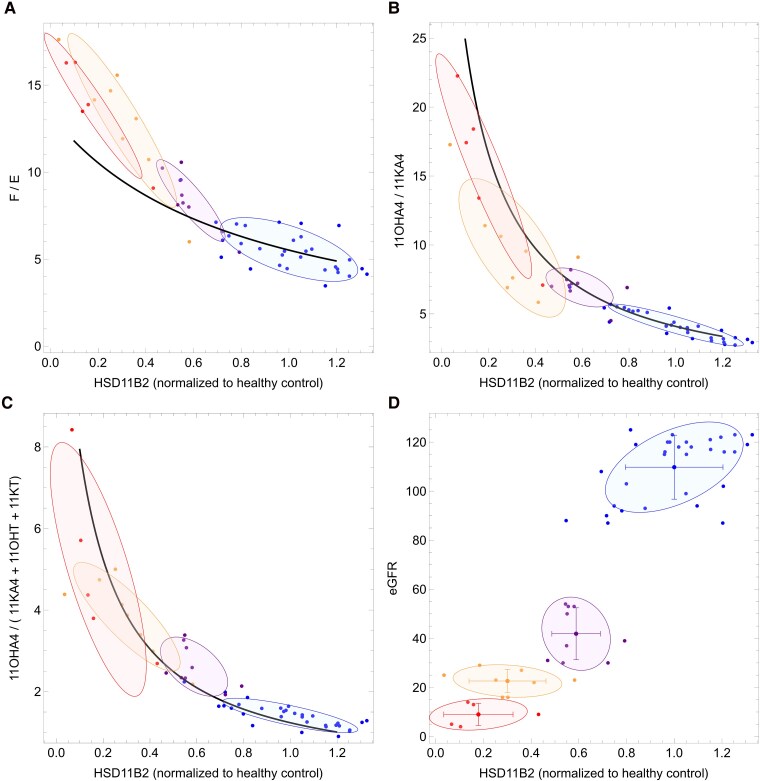
Computational modeling reveals reduced 11β-hydroxysteroid dehydrogenase type 2 (HSD11B2) activity as the causative factor in reduced 11-oxygenated androgen biosynthesis in chronic kidney disease (CKD). Ratios of measured steroids are plotted against estimated HSD11B2 expression levels in (A) F/E, (B) 11OHA4/11KA4, and (C) 11OHA4/(11KA4 + 11KT +11OHT). (D) Shows the correlation of the estimated HSD11B2 expression with estimated glomerular filtration rate (eGFR). Data points are color coded according to disease stage: blue, control participants; purple, CKD stage 3; orange, CKD stage 4; red, CKD stage 5. Each data point represents a specific individual. The large dots with error in (D) represent the mean and standard error for each disease stage. The shaded ellipses represent the covariance between the model and data. The solid lines in A-C show the models prediction on the effect of HSD11B2 expression on serum steroid ratio.

We then used the model to explore how changes in the expression level of each individual enzyme could account for the measured changes in serum steroid levels in the patients with CKD, while keeping the other enzymes at reference expression level. Our simulations revealed that a decrease in the relative expression of HSD11B2 alone could account for the altered serum steroid profiles, and that the model fits for HSD11B2 were better than for individual perturbations of any of the other 3 enzymes. The model simulations of reduced relative HSD11B2 expression for F/E, 11OHA4/11KA4, and 11OHA4/(11KA4 + 11KT + 11OHT) ratios are shown in Fig. 5A-5C. For these simulations, the expression values of the 4 enzymes are set to the mean values of the healthy control group, and the effect of varying the expression of HSD11B2 between 0.1 and 1.2 times this expression level (while keeping the other enzymes at the control values) on the steady-state steroid concentrations were analyzed. In addition, the figures show the data of individual patients, together with the covariance error between the data (error ellipse). The figures confirm the central role played by HSD11B2 in the peripheral metabolism of both glucocorticoids and 11-oxygenated androgens. By setting the expression levels of the enzymes to the levels in the healthy control group, and only varying HSD11B2 expression, the model quite accurately describes the different disease states. In addition, the fitted relative HSD11B2 expression levels combined with the serum steroid ratios formed clear groupings for each disease stage (Fig. 5A-5C). By combining the model prediction of relative HSD11B2 expression levels with clinically measured eGFR, we were able to identify distinct clusters for each disease stage, thus demonstrating the power of the modeling approach and confirming the key role of HSD11B2 in controlling 11-oxygenated androgen generation (Fig. 5D).

### Correlation of Serum 11-Oxygenated Androgens With Markers of Body Composition and Anemia in CKD

There was no significant correlation noted between body composition parameters and serum 11-oxygenated androgen concentrations in patients with CKD or controls. When stratified by sex, there was also no correlation between the ratio of 11OHA4/(11KA4 + 11OHT + 11KT) and fat-free mass, BMI, visceral fat mass, muscle, or fat percentage (Table S2 (33)). There was a positive association between hemoglobin and 11KT in the CKD cohort (Spearman r = 0.64, *P* < .0001). This relationship was present in both sexes (female Spearman r = 0.72, *P* < .0001, male Spearman r = 0.63, *P* < .0001) and persisted when accounting for eGFR (r^2^ = 0.56, *P* < .0001, Table S3 (33)).

## Discussion

This study characterizes, for the first time, the pivotal role played in vivo by HSD11B2 in human 11-oxygenated androgen biosynthesis. Previous in vitro data have suggested a key role for HSD11B2 in the biosynthesis of the potent 11-oxygenated androgen 11KT (11, 12, 36). This includes the observation that 11OHA4 is the primary 11-oxygenated androgen precursor produced by the human adrenal, but it requires further peripheral downstream activation (11, 37). 11OHA4 is not itself a substrate for the key androgen-activating enzyme AKR1C3, and as such must be converted to 11KA4 prior to the AKR1C3-mediated production of 11KT (15, 16). While previous studies have demonstrated the conversion of 11OHA4 to 11KA4 in vitro, the importance of this reaction and the contribution of renal HSD11B2 has not been demonstrated in vivo (12, 13). Herein, we confirm a clear role for renal HSD11B2 in the peripheral biosynthesis of 11-oxygenated androgens and further confirm the kidney as a major site of HSD11B2 expression and activity. It is unclear if other HSD11B2-expressing tissues contribute to 11-oxygenated androgen biosynthesis. However, given the global decline in 11-oxygenated androgen concentrations observed in our CKD cohort, the contributions of other HSD11B2-expressing tissues are likely to be marginal. Utilizing a cohort of patients with CKD as a human in vivo model of impaired HSD11B2 activity, we show that downstream activation of adrenal-derived 11OHA4 to 11KA4, 11KT, and 11OHT is reduced in parallel with the decline of eGFR and HSD11B2 activity. Using a computational modeling approach, we were able to demonstrate that reduced HSD11B2 expression is the central enzymatic perturbation driving dysfunctional 11-oxygenated androgen biosynthesis in this cohort. Notably, circulating classic androgens are relatively preserved in the setting of impaired renal function.

We have corroborated the findings of previous studies by demonstrating impaired HSD11B2 activity in glucocorticoid inactivation in patients with CKD. This is reflected by an elevated F/E ratio in both serum and urine of patients with CKD compared with control participants. HSD11B2 activity demonstrated a positive correlation with eGFR, with a step-wise reduction in HSD11B2 activity as eGFR declines across each stage of CKD. This validates the findings of previous studies focused on glucocorticoid metabolism in CKD (22, 23, 38). Quinkler et al previously demonstrated reduced mRNA expression of HSD11B2 in renal biopsies of 95 patients with CKD, alongside an attenuated HSD11B2 immunofluorescence signal on kidney biopsy specimens of 5 patients with CKD compared with 5 healthy controls, demonstrating that reduced HSD11B2 activity is due to decreased enzyme expression (22). In the present study on 11-oxygenated androgen biosynthesis in CKD, we have demonstrated significantly reduced serum concentrations of the downstream metabolites 11KA4, 11KT, and 11OHT in patients with CKD. In patients with CKD, 11OHA4, which is biochemically inert, constitutes a median of 77% of the circulating 11-oxygenated androgen pool compared with only 60% in healthy controls. The change in 11OHA4% of the total 11-oxygenated androgen pool indicates a build-up of precursor steroid due to impaired HSD11B2 activity. Conversely, the relative proportion of the potent androgen 11KT is reduced in patients with CKD, contributing only 7% of the circulating pool of 11-oxygenated androgens compared with 17% in controls. This validates the central importance of renal HSD11B2 activity in 11-oxygenated androgen activation, providing definitive in vivo biochemical insights into peripheral 11-oxygenated androgen biosynthesis.

Previous studies have identified 11OHA4 as the predominant 11-oxygenated androgen of adrenal origin, with 11KA4, 11KT, and 11OHT reliant on peripheral conversion by enzymes HSD11B2, AKR1C3, and HSD11B1, respectively (3, 10, 11, 15, 37). Our data highlight that downstream generation of both 11KT and 11OHT is significantly impaired in the absence of efficient conversion of 11OHA4 to 11KA4 by HSD11B2 due to a reduced pool of 11KA4 for peripheral activation. Intriguingly, the observation that 11OHT levels decline significantly with CKD stage confirms that the majority of circulating 11OHT is derived via peripheral conversion from 11KT by HSD11B1 rather than directly from the adrenal gland (11, 15). Reduced HSD11B2 activity will also limit the conversion of peripherally derived 11OHT back to 11KT. This impacts on the conversion of the already reduced levels of adrenal-derived 11OHT to 11KT, thus contributing to an overall decrease in circulating 11KT. It should be noted, however, that the direct HSD11B2-mediated conversion of 11OHT to 11KT may play a larger role in congenital adrenal hyperplasia, where the adrenal output of 11OHT is significantly increased (8, 11).

Using a computational model derived from the parameterized rate equations of key enzymes involved in peripheral 11-oxygenated androgen biosynthesis, we were able to demonstrate that decreased HSD11B2 activity alone could account for changes in the serum steroid profiles in patients with CKD, and that the predicted decrease in relative HSD11B2 correlated well with disease stage (with only a small underestimation for the F/E ratio in the severest disease stage, Fig 5A). The model simultaneously accounted for the known changes in peripheral glucocorticoid metabolism associated with CKD, as well as reduced 11-oxygenated androgen activation as reported here for the first time, thus demonstrating the key role played by HSD11B2 in both steroid classes. Significantly, the combination of the relative HSD11B2 expression levels predicted by the model from the serum steroids and the clinically determined eGFR resulted in distinct clusters for each disease stage. This not only demonstrated the power of the modeling approach, but also illustrates its potential utility as a sensitive tool for monitoring CKD disease progression in the future following further validation with larger cohorts. Given the rising prevalence mortality and morbidity of CKD (39-43), identifying novel insights into disease pathophysiology may lead to improved therapeutic strategies in reducing the burden of CKD-related morbidity.

The clinical significance of 11-oxygenated androgen excess and deficiency in human health and disease remains poorly understood. However, 11-oxygenated androgens are the predominant androgens detected in multiple disorders of androgen excess, including polycystic ovary syndrome, congenital adrenal hyperplasia, premature adrenarche, and Cushing syndrome (2, 3, 6, 7) as well as physiologically in women after menopause (4, 5). Particularly in postmenopausal women, 11-oxygenated androgens are the most abundant source of active androgens (4). Turcu et al described reduced 11-oxygenated androgens in patients with primary adrenal insufficiency (8). In this study we have demonstrated that the total pool of 11-oxygenated androgens remains relatively unchanged indicating consistent adrenal output, but that the distribution of the 11-oxygenated androgens changed significantly and that these changes are proportional to the severity of kidney disease.

The clinical consequences, if any, of reduced 11-oxygenated androgen activation on general health and well-being in patients with CKD remain unknown. As 11KT is a potent androgen receptor agonist (12), 11-oxygenated androgen deficiency may potentially contribute to the development of sarcopenia, frailty, and overall mortality particularly in female patients with CKD. Previous studies have shown that 11-oxygenated androgen concentrations do not decline with age (4, 5), and might theoretically continue to confer androgenic health benefits on muscle strength with ageing. Certainly, tissue-specific regulation of adrenal steroids may affect lean muscle mass with previous reports of increased lean muscle mass reported in HSD11B1 inhibition (44). Our group have recently shown that HSD11B1 inhibition leads to a significant increase in 11KT and, therefore, the improvements noted in lean muscle mass may be attributed to favorable androgenic effects of 11-oxygenated androgens (15, 44). Conversely, we hypothesize that reduced HSD11B2 activity may contribute to reductions in lean muscle mass and sarcopenia in patients with CKD. In the current study, we did not find any definitive correlation between 11-oxygenated androgen biosynthesis and surrogate markers of body composition. However, we did identify a positive relationship between hemoglobin and 11KT, independent of eGFR, which suggests that 11-oxygenated androgen deficiency could be a predictor of or a contributor to anemia in CKD. Future larger studies are required to uncover the clinical consequences of reduced 11-oxygenated androgens in CKD.

Hypogonadism can occur in both sexes in CKD and is attributed to uremia-induced loss of gonadotropin-releasing hormone pulsatility, chronic stress, and hyperprolactinemia (45). The prevalence of hypogonadism in males with CKD has been reported to be as high as 40% to 60% in some cohorts (46-48) and is associated with an independent 2- to 3-fold increase in cardiovascular and all-cause mortality (48, 49). However T concentrations were not significantly reduced in our CKD cohort. In contrast, potent 11-oxygenated androgens were significantly reduced, even in moderate CKD. We would therefore postulate that loss of 11-oxygenated androgens in CKD may be a greater differential contributor to adverse outcomes in CKD, and could represent a potential novel therapeutic target. We identified reduced concentrations of both the classic androgen precursors DHEA and A4 in patients with CKD in both sexes in the present study compared with the Beaumont control cohort. However, this observation is much more likely to represent the significant age difference between the cohorts rather than a biological finding primarily driven by renal impairment. Indeed when compared against the more age-matched Birmingham cohort and in regression models, this observation was lost.

One of the limitations of our study is the discrepancy in age and sex distribution between the CKD and Beaumont control populations. To account for this, we compared results against a much larger age-matched control cohort from the University of Birmingham. In tandem with this, we performed multiple linear regression and demonstrated the persistence of a significant, although weak, association between renal function and 11KA4, 11KT, and 11OHT after adjustment of the model for age, sex, and BMI. Additionally we demonstrated a positive association between 11OHA4 with BMI in our cohort. In healthy populations, previous studies have demonstrated a positive relationship between BMI and 11KT, potentially due to increased AKR1C3 activity in adipose tissue (5, 32). 11OHT also shared an association with age in our cohort which has previously been shown in a healthy control population; however, in this previous study the association was lost once adjusted for BMI (5). A further potential limitation is that urinary glucocorticoid and adrenal androgen metabolite excretion were globally reduced in patients with CKD compared with the Beaumont control cohort, especially in more severe disease (stages 4 and 5). It is unclear whether the urinary steroid metabolome is altered in patients with severe renal dysfunction, and, therefore, analysis of serum steroids may be a more accurate approach to study steroid metabolism in this cohort.

The emergence of 11-oxygenated androgens as potent androgens contributing significantly to disease pathogenesis has reignited research interest in adrenal steroid biosynthesis (2, 3). Here we have clearly identified in vivo that HSD11B2 is integral to the biosynthesis and activation of 11-oxygenated androgens. The clinical consequences of reduced HSD11B2 activity resulting in reduced 11-oxygenated androgen concentrations in patients with CKD remain to be elucidated and should be the focus of future research endeavors.

## Data Availability

The data underlying this article cannot be shared publicly, as participants of this study did not agree for their data to be shared publicly. Anonymized data may be made available to external researchers upon reasonable request.

## Abbreviations

11βOHAn: 11β-hydroxyandrosterone
11KA4: 11-ketoandrostenedione
11KT: 11-ketotestosterone
11OHA4: 11β-hydroxyandrostenedione
11OHT: 11β-hydroxytestosterone
11-oxoAn: 11-oxoandrosterone
A4: androstenedione
AKR1C3: aldo-keto reductase type 1C3
BMI: body mass index
CKD: chronic kidney disease
DHT: 5α-dihydrotestosterone
E: cortisone
eGFR: estimated glomerular filtration rate
F: cortisol
DHEA: dehydroepiandrosterone
HSD11B1: 11β-hydroxysteroid dehydrogenase type 1
HSD11B2: 11β-hydroxysteroid dehydrogenase type 2
HSD17B2: 17β-hydroxysteroid dehydrogenase type 2
T: testosterone

## References

[1] Turcu AF, Rege J, Auchus RJ, Rainey WE. 11-Oxygenated androgens in health and disease. Nat Rev Endocrinol. 2020;16(5):284‐296. 32203405 10.1038/s41574-020-0336-x PMC7881526

[2] Turcu AF, Auchus RJ. Clinical significance of 11-oxygenated androgens. Curr Opin Endocrinol Diabetes Obes. 2017;24(3):252‐259. 28234803 10.1097/MED.0000000000000334 PMC5819755

[3] Storbeck KH, O'Reilly MW. The clinical and biochemical significance of 11-oxygenated androgens in human health and disease. Eur J Endocrinol 2023;188(4):R98‐R109. doi:10.1093/ejendo/lvad047 37041725

[4] Nanba AT, Rege J, Ren J, Auchus RJ, Rainey WE, Turcu AF. 11-oxygenated C19 steroids do not decline with age in women. J Clin Endocrinol Metab. 2019;104(7):2615‐2622. 30753518 10.1210/jc.2018-02527 PMC6525564

[5] Schiffer L, Kempegowda P, Sitch AJ, et al Classic and 11-oxygenated androgens in serum and saliva across adulthood: a cross-sectional study analyzing the impact of age, body mass index, and diurnal and menstrual cycle variation. Eur J Endocrinol. 2023;188(1):86‐100. 10.1093/ejendo/lvac017 36651154

[6] O'Reilly MW, Kempegowda P, Jenkinson C, et al 11-oxygenated C19 steroids are the predominant androgens in polycystic ovary syndrome. J Clin Endocrinol Metab. 2017;102(3):840‐848. 27901631 10.1210/jc.2016-3285 PMC5460696

[7] Kamrath C, Wettstaedt L, Boettcher C, Hartmann MF, Wudy SA. Androgen excess is due to elevated 11-oxygenated androgens in treated children with congenital adrenal hyperplasia. J Steroid Biochem Mol Biol. 2018;178:221‐228. 29277706 10.1016/j.jsbmb.2017.12.016

[8] Turcu AF, Nanba AT, Chomic R, et al Adrenal-derived 11-oxygenated 19-carbon steroids are the dominant androgens in classic 21-hydroxylase deficiency. Eur J Endocrinol. 2016;174(5):601‐609. 26865584 10.1530/EJE-15-1181 PMC4874183

[9] Barnard M, Mostaghel EA, Auchus RJ, Storbeck KH. The role of adrenal derived androgens in castration resistant prostate cancer. J Steroid Biochem Mol Biol. 2020;197:105506. 31672619 10.1016/j.jsbmb.2019.105506 PMC7883395

[10] Pretorius E, Arlt W, Storbeck K-H. A new dawn for androgens: novel lessons from 11-oxygenated C19 steroids. Mol Cell Endocrinol. 2017;441:76‐85. 27519632 10.1016/j.mce.2016.08.014

[11] Storbeck KH . A commentary on the origins of 11-ketotestosterone. Eur J Endocrinol. 2022;187(6):C5‐C8. 36173704 10.1530/EJE-22-0820

[12] Storbeck KH, Bloem LM, Africander D, Schloms L, Swart P, Swart AC. 11β-Hydroxydihydrotestosterone and 11-ketodihydrotestosterone, novel C19 steroids with androgenic activity: a putative role in castration resistant prostate cancer? Mol Cell Endocrinol. 2013;377(1-2):135‐146. 23856005 10.1016/j.mce.2013.07.006

[13] Gent R, du Toit T, Bloem LM, Swart AC. The 11β-hydroxysteroid dehydrogenase isoforms: pivotal catalytic activities yield potent C11-oxy C(19) steroids with 11βHSD2 favouring 11-ketotestosterone, 11-ketoandrostenedione and 11-ketoprogesterone biosynthesis. J Steroid Biochem Mol Biol. 2019;189:116‐126. 30825506 10.1016/j.jsbmb.2019.02.013

[14] Zheng J, Wang Y, Jiang J, et al Decoding 11-oxygenated androgen synthesis: insights from enzyme gene expression and LC—MS/MS quantification. Eu J Endocrinol. 2024;191(3):288‐299. 10.1093/ejendo/lvae104 39219353

[15] Schiffer L, Oestlund I, Snoep JL, et al Inhibition of the glucocorticoid-activating enzyme 11β-hydroxysteroid dehydrogenase type 1 drives concurrent 11-oxygenated androgen excess. FASEB J. 2024;38(7):e23574. 38551804 10.1096/fj.202302131R

[16] Barnard M, Quanson JL, Mostaghel E, Pretorius E, Snoep JL, Storbeck KH. 11-Oxygenated androgen precursors are the preferred substrates for aldo-keto reductase 1C3 (AKR1C3): implications for castration resistant prostate cancer. J Steroid Biochem Mol Biol. 2018;183:192‐201. 29936123 10.1016/j.jsbmb.2018.06.013 PMC6283102

[17] Pretorius E, Africander DJ, Vlok M, Perkins MS, Quanson J, Storbeck KH. 11-Ketotestosterone and 11-ketodihydrotestosterone in castration resistant prostate cancer: potent androgens which can No Longer be ignored. PLoS One. 2016;11(7):e0159867. 27442248 10.1371/journal.pone.0159867 PMC4956299

[18] Chen TK, Knicely DH, Grams ME. Chronic kidney disease diagnosis and management: a review. JAMA. 2019;322(13):1294‐1304. 31573641 10.1001/jama.2019.14745 PMC7015670

[19] Dineen R, Stewart PM, Sherlock M. Factors impacting on the action of glucocorticoids in patients receiving glucocorticoid therapy. Clin Endocrinol (Oxf). 2019;90(1):3‐14. 30120786 10.1111/cen.13837

[20] Quinkler M, Stewart PM. Hypertension and the cortisol-cortisone shuttle. J Clin Endocrinol Metab. 2003;88(6):2384‐2392. 12788832 10.1210/jc.2003-030138

[21] Chapman K, Holmes M, Seckl J. 11β-hydroxysteroid dehydrogenases: intracellular gate-keepers of tissue glucocorticoid action. Physiol Rev. 2013;93(3):1139‐1206. 23899562 10.1152/physrev.00020.2012 PMC3962546

[22] Quinkler M, Zehnder D, Lepenies J, et al Expression of renal 11beta-hydroxysteroid dehydrogenase type 2 is decreased in patients with impaired renal function. Eur J Endocrinol. 2005;153(2):291‐299. 16061836 10.1530/eje.1.01954

[23] N'Gankam V, Uehlinger D, Dick B, Frey BM, Frey FJ. Increased cortisol metabolites and reduced activity of 11beta-hydroxysteroid dehydrogenase in patients on hemodialysis. Kidney Int. 2002;61(5):1859‐1866. 11967038 10.1046/j.1523-1755.2002.00308.x

[24] Mongia A, Vecker R, George M, et al Role of 11βHSD type 2 enzyme activity in essential hypertension and children with chronic kidney disease (CKD). J Clin Endocrinol Metab. 2012;97(10):3622‐3629. 22872687 10.1210/jc.2012-1411

[25] McQuarrie EP, Freel EM, Mark PB, Fraser R, Connell JMC, Jardine AG. Urinary sodium excretion is the main determinant of mineralocorticoid excretion rates in patients with chronic kidney disease. Nephrol Dial Transplantat. 2013;28(6):1526‐1532. 10.1093/ndt/gft007 23413088

[26] Sagmeister MS, Taylor AE, Fenton A, et al Glucocorticoid activation by 11β-hydroxysteroid dehydrogenase enzymes in relation to inflammation and glycaemic control in chronic kidney disease: a cross-sectional study. Clin Endocrinol (Oxf). 2019;90(1):241‐249. 30358903 10.1111/cen.13889 PMC6334281

[27] Inker LA, Eneanya ND, Coresh J, et al New creatinine- and cystatin C–based equations to estimate GFR without race. N Engl J Med. 2021;385(19):1737‐1749. 34554658 10.1056/NEJMoa2102953 PMC8822996

[28] Kidney Disease: Improving Global Outcomes (KDIGO) CKD Work Group . KDIGO 2024 clinical practice guideline for the evaluation and management of chronic kidney disease. Kidney Int. 2024;105(4):S117‐S314. 38490803 10.1016/j.kint.2023.10.018

[29] Schiffer L, Shaheen F, Gilligan LC, et al Multi-steroid profiling by UHPLC-MS/MS with post-column infusion of ammonium fluoride. J Chromatogr B Analyt Technol Biomed Life Sci. 2022;1209:123413. 10.1016/j.jchromb.2022.123413 35988498

[30] Bancos I, Taylor AE, Chortis V, et al Urine steroid metabolomics for the differential diagnosis of adrenal incidentalomas in the EURINE-ACT study: a prospective test validation study. Lancet Diabetes Endocrinol. 2020;8(9):773‐781. 32711725 10.1016/S2213-8587(20)30218-7 PMC7447976

[31] Shackleton CH . Mass spectrometry in the diagnosis of steroid-related disorders and in hypertension research. J Steroid Biochem Mol Biol. 1993;45(1-3):127‐140. 8481337 10.1016/0960-0760(93)90132-g

[32] Davio A, Woolcock H, Nanba AT, et al Sex differences in 11-oxygenated androgen patterns across adulthood. J Clin Endocrinol Metab. 2020;105(8):e2921‐e2929. 32498089 10.1210/clinem/dgaa343 PMC7340191

[33] Tomkins M, McDonnell T, Cussen L, et al Supplementary material for Impaired 11β-hydroxysteroid dehydrogenase type 2 activity in kidney disease disrupts 11-oxygenated androgen biosynthesis. 2024. *Figshare.* 10.6084/m9.figshare.27043891 PMC12086407 39382395

[34] Oestlund I, Snoep J, Schiffer L, Wabitsch M, Arlt W, Storbeck KH. The glucocorticoid-activating enzyme 11β-hydroxysteroid dehydrogenase type 1 catalyzes the activation of testosterone. J Steroid Biochem Mol Biol. 2024;236:106436. 38035948 10.1016/j.jsbmb.2023.106436

[35] Institute of Inflammation and Aging, University of Birmingham . The Birmingham 1000 Elders group. Accessed August 28, 2023. https://www.birmingham.ac.uk/research/inflammation-ageing/research/1000-elders/elders.aspx

[36] Swart AC, Schloms L, Storbeck K-H, et al 11β-Hydroxyandrostenedione, the product of androstenedione metabolism in the adrenal, is metabolized in LNCaP cells by 5α-reductase yielding 11β-hydroxy-5α-androstanedione. J Steroid Biochem Mol Biol. 2013;138:132‐142. 23685396 10.1016/j.jsbmb.2013.04.010

[37] Rege J, Nakamura Y, Satoh F, et al Liquid chromatography-tandem mass spectrometry analysis of human adrenal vein 19-carbon steroids before and after ACTH stimulation. J Clin Endocrinol Metab. 2013;98(3):1182‐1188. 23386646 10.1210/jc.2012-2912 PMC3590473

[38] Gant CM, Minovic I, Binnenmars H, et al Lower renal function is associated with derangement of 11-β hydroxysteroid dehydrogenase in type 2 diabetes. J Endocr Soc. 2018;2(7):609‐620. 29942925 10.1210/js.2018-00088 PMC6007243

[39] Nowak N, Mellotte G, O'Halloran A, Kenny R, Sexton D. Chronic Kidney Disease in community-dwelling adults aged 50+ years in Ireland: A Report from TILDA and the National Renal Office 10/01 2023.

[40] Hill NR, Fatoba ST, Oke JL, et al Global prevalence of chronic kidney disease—a systematic review and meta-analysis. PLoS One. 2016;11(7):e0158765. 27383068 10.1371/journal.pone.0158765 PMC4934905

[41] Kovesdy CP . Epidemiology of chronic kidney disease: an update 2022. Kidney Int Suppl (2011). 2022/04/01/2022;12(1):7‐11. 10.1016/j.kisu.2021.11.003 PMC9073222 35529086

[42] GBD Chronic Kidney Disease Collaboration . Global, regional, and national burden of chronic kidney disease, 1990-2017: a systematic analysis for the Global Burden of Disease Study 2017. The Lancet. 2020;395(10225):709‐733. 10.1016/S0140-6736(20)30045-3 PMC7049905 32061315

[43] Foreman KJ, Marquez N, Dolgert A, et al Forecasting life expectancy, years of life lost, and all-cause and cause-specific mortality for 250 causes of death: reference and alternative scenarios for 2016-40 for 195 countries and territories. Lancet. 2018;392(10159):2052‐2090. 30340847 10.1016/S0140-6736(18)31694-5 PMC6227505

[44] Hardy RS, Botfield H, Markey K, et al 11βHSD1 inhibition with AZD4017 improves lipid profiles and lean muscle mass in idiopathic intracranial hypertension. J Clin Endocrinol Metab. 2021;106(1):174‐187. 33098644 10.1210/clinem/dgaa766 PMC7765633

[45] Kaka N, Sethi Y, Patel N, et al Endocrine manifestations of chronic kidney disease and their evolving management: a systematic review. Dis Mon. 2022;68(12):101466. 35965104 10.1016/j.disamonth.2022.101466

[46] Garibotto G, Esposito P, Picciotto D, Verzola D. Testosterone disorders and male hypogonadism in kidney disease. Semin Nephrol. 2021;41(2):114‐125. 34140090 10.1016/j.semnephrol.2021.03.006

[47] Albaaj F, Sivalingham M, Haynes P, et al Prevalence of hypogonadism in male patients with renal failure. Postgrad Med J. 2006;82(972):693‐696. 17068282 10.1136/pgmj.2006.045963 PMC2653914

[48] Carrero JJ, Qureshi AR, Parini P, et al Low serum testosterone increases mortality risk among male dialysis patients. J Am Soc Nephrol. 2009;20(3):613‐620. 19144759 10.1681/ASN.2008060664 PMC2653676

[49] Kyriazis J, Tzanakis I, Stylianou K, et al Low serum testosterone, arterial stiffness and mortality in male haemodialysis patients. Nephrol Dial Transplant. 2011;26(9):2971‐2977. 21427069 10.1093/ndt/gfq847

